# Electronic Health Literacy Among Baby Boomers: A Typology

**DOI:** 10.3928/24748307-20231213-02

**Published:** 2024-01

**Authors:** Lynn Sudbury-Riley, Mary FitzPatrick, Peter J. Schulz, Alexandra Hess

## Abstract

**Background:**

Forecasts suggest that older adults will place unprecedented demands on future health care systems. Electronic health (eHealth) resources can potentially mitigate some pressures, but to be effective patients need to be able to use them. The negative relationship between eHealth literacy and age usually results in older adults classified as one homogenous mass, which misses the opportunity to tailor interventions.

**Objective:**

This research examines similarities and differences within the baby boom cohort among a sample that uses the internet for health information.

**Methods:**

We used an electronic survey with random samples of baby boomers (*N* = 996) from the United States, the United Kingdom, and New Zealand.

**Key Results:**

Four distinct subgroups, or segments, emerged. While not different from a socioeconomic perspective, these four groups have very different levels of eHealth literacy and corresponding health behaviors. Therefore, we contribute a more complex picture than is usually presented in eHealth studies.

**Conclusions:**

Resulting insights offer a useful starting point for providers wishing to better tailor health products, services, and communications to this large cohort of future older individuals. [***HLRP: Health Literacy Research and Practice*. 2024;8(1):e3–e11.**]

## Abstract (plain language summary)

Plain Language Summary:

We researched the ability to find and use eHealth information among an international cohort of baby boomers. We identified four distinct groups whose use of ehealth differs considerably from each other. Findings give guidance to health care providers on how they can work with the different groups to encourage better health care management.

Health literacy is the capacity to obtain, process, and understand health information for decision-making (Zaim et al., 2021). The coronavirus disease 2019 (COVID-19) pandemic spotlighted its importance, while the COVID-19 infodemic underscored global health literacy problems (Paakkari & Okan, 2020). Low health literacy is associated with poor health outcomes including recognizing symptoms, seeking services, understanding advice, and increased mortality (Chakkalakal et al., 2017; Chang et al., 2020; Griffeth et al., 2022; Nandyala et al., 2018). Adequate health literacy is key to actively managing one's own health (Muvuka et al., 2020), impacts patient-provider interactions (Gibson et al., 2022), and ultimately leads to fewer hospitalizations and reduced costs (Conard, 2019).

Increasingly, involving patients in health management is pursued via digital resources (Harris et al., 2019). Consequently, electronic health (eHealth) is progressively important due to a surging reliance on technology to engage with health information and services (Petrakaki et al., 2018; Zaim et al., 2021). eHealth literacy encompasses “the ability to seek, find, understand, and appraise health information from electronic sources and apply the knowledge gained to addressing or solving a health problem” (Norman & Skinner, 2006, p. e9). These elements are important because in addition to excellent health information, the Internet contains much misinformation, some of it potentially harmful (Wagner et al., 2022). Yet, Google receives approximately 1 billion health questions every day (Drees, 2019), with average searches increasing prior to a hospital visit (Asch et al., 2019).

eHealth literacy and age are negatively related, even after controlling for education and general health literacy (Hsu, 2019). This is important because of population ageing globally. The number of persons age 80 years or older is projected to triple from 143 million in 2019 to 426 million by 2050 (United Nations, 2019). The profundity of this demographic change suggests future strains on health care systems; marked increases in age-related diseases (e.g., arthritis, osteoporosis, type 2 diabetes, cardiovascular disease) are predicted (Guzman-Castillo et al., 2017). eHealth has the potential to enhance patient empowerment and participation (World Health Organization [WHO], 2020), mirroring the paradigm shift away from passive patients to personalized care. However, achieving the benefits of personalized care requires knowledge and skills to collaborate (Royal College of Physicians, 2018), and although older adults are increasingly using eHealth resources (Hung et al., 2020), they lag behind younger generations (Hsu, 2019).

Operationalization of true personalized care is impracticable because unique care packages for everyone are unrealistic to resource (Chong et al., 2019). Behavioral and psychographic segmentation could help this problem. Segmentation identifies population subgroups that differ meaningfully from each other, while displaying homogenous key needs or behaviors (Elrod & Fortenberry, 2018). Segmentation enhances effective resource allocation by focusing resources where needed (Dibb, 1999). Patient segmentation, however, tends to focus on clinical conditions or practitioner appraisals of requirements, often failing to consider comorbidity, or contemplate different needs within segments, causing fragmentation of services and resource inefficiency (Eissens van der Laan et al., 2014). In contrast, psychographic segmentation utilizes the actions, preferences, and beliefs of service users for deeper understanding of behavior and requirements, providing a strategic foundation for better tailoring of products, services, communications, and required interventions (Koubaa et al., 2017).

Few studies examine health segmentation among ageing populations. Eissens van der Laan et al. (2014) segmented older Dutch adults (age 65 years and older) based on biopsychosocial functioning, identifying five homogenous groups. Lafortune et al. (2009) found four segments of older Canadians (age 64 years and older) differentiated on health and service use. Neither study incorporated eHealth. Research focusing on eHealth among older adults tends to concentrate on the drivers and barriers associated with using various health technologies (Huvila et al., 2022; Pywell et al., 2020), or sociodemographic differences between users and non-users (Tennant et al., 2015). However, we know relatively little about eHealth patterns and different behaviors within populations of older adults who do use the internet for health purposes. Choi and Dinitto (2013) identified affordability as a reason why some individuals who are older than age 60 years had discontinued use. Others suggest eHealth literacy is associated with ownership or access to electronic devices (Nguyen et al., 2017) or with levels of technology reluctance (e.g., feelings of intimidation, anxiety, computer stress, or trust) (Arcury et al. 2020; Meng et al., 2022; Vroman et al., 2015). A small amount of research examines attitudes toward reliance on clinicians for decision-making (Arcury et al., 2020). None, however, applies the concept of segmentation.

Consequently, we examine eHealth literacy and related behaviors among baby boomers: the cohort born between 1946 and 1964 in three disparate nations—the United Kingdom, the United States, and New Zealand. Across all three countries, projections suggest unprecedented demands on future health care systems (Clement, 2021; King et al., 2013; Spoonley, 2020). Specifically, we aim to address the following questions:

1. Are eHealth literate segments identifiable among baby boomers who use the internet for eHealth?
2. Which information sources do they access and why?
3. How are health behaviors and practitioner relationships informed by health information?
4. Do further key psychographic variables differentiate these segments?

Answers to these questions offer a strategic starting point to improved planning, delivery of personalized care, and the development of interventions to better prepare for the future needs of this important cohort.

## Methods

### Instrument Development

An extensive eHealth literature preceded using a snowball sample of New Zealand baby boomers (*n* = 24) to run 3 focus groups and 8 semi-structured interviews probing use of eHealth technologies. These are established procedures for generating a comprehensive data collection instrument (Boateng et al., 2018). The instrument comprised questions about health, sociodemographics, and a range of potentially useful scales and items, which are detailed in **Table A**.

**Table A:**
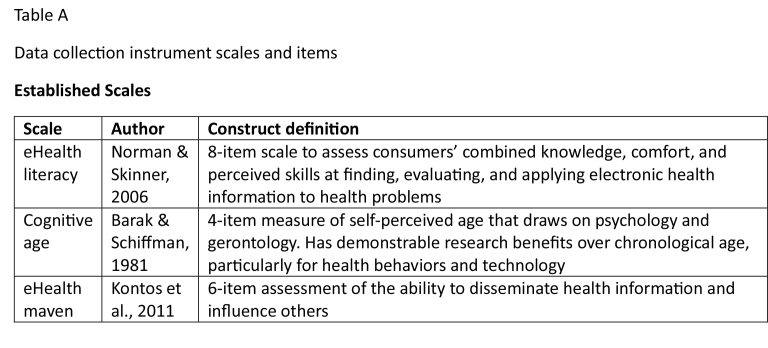
Data collection instrument scales and items

**Established Scales**		
**Scale**	**Author**	**Construct definition**
eHealth literacy	Norman & Skinner, 2006	8-item scale to assess consumers' combined knowledge, comfort, and perceived skills at finding, evaluating, and applying electronic health information to health problems
Cognitive age	Barak & Schiffman, 1981	4-item measure of self-perceived age that draws on psychology and gerontology. Has demonstrable research benefits over chronological age, particularly for health behaviors and technology
eHealth maven	Kontos et al., 2011	6-item assessment of the ability to disseminate health information and influence others

**Table:**
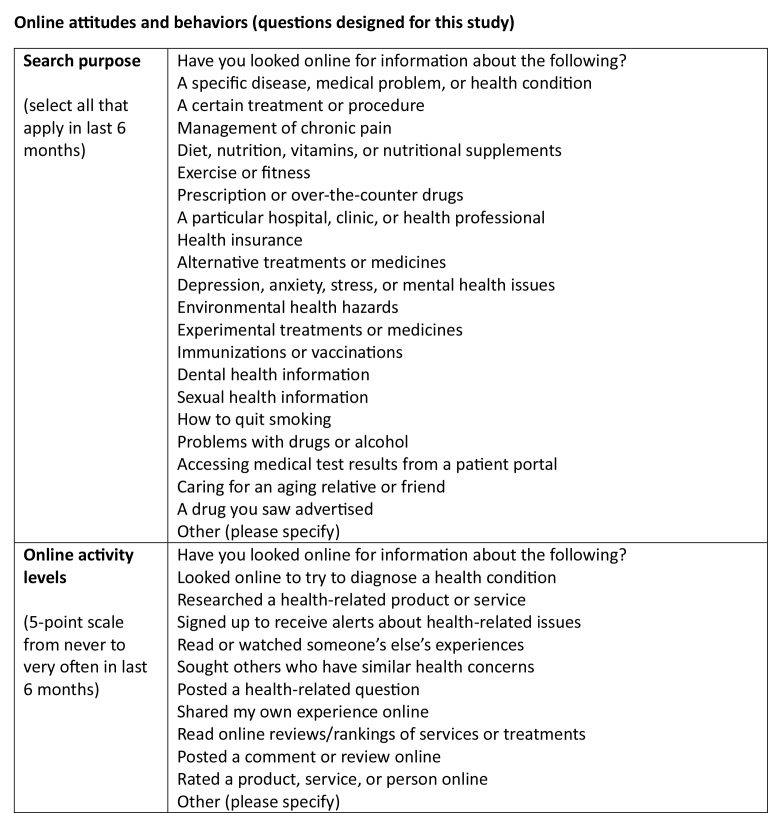


**Online attitudes and behaviors (questions designed for this study)**	
**Search purpose **(select all that apply in last 6 months)	Have you looked online for information about the following?
	A specific disease, medical problem, or health condition
	A certain treatment or procedure
	Management of chronic pain
	Diet, nutrition, vitamins, or nutritional supplements
	Exercise or fitness
	Prescription or over-the-counter drugs
	A particular hospital, clinic, or health professional
	Health insurance
	Alternative treatments or medicines
	Depression, anxiety, stress, or mental health issues
	Environmental health hazards
	Experimental treatments or medicines
	Immunizations or vaccinations
	Dental health information
	Sexual health information
	How to quit smoking
	Problems with drugs or alcohol
	Accessing medical test results from a patient portal
	Caring for an aging relative or friend
	A drug you saw advertised
	Other (please specify)

**Online activity levels **(5-point scale from never to very often in last 6 months)	Have you looked online for information about the following?
	Looked online to try to diagnose a health condition
	Researched a health-related product or service
	Signed up to receive alerts about health-related issues
	Read or watched someone's else's experiences
	Sought others who have similar health concerns
	Posted a health-related question
	Shared my own experience online
	Read online reviews/rankings of services or treatments
	Posted a comment or review online
	Rated a product, service, or person online
	Other (please specify)

**Table:**
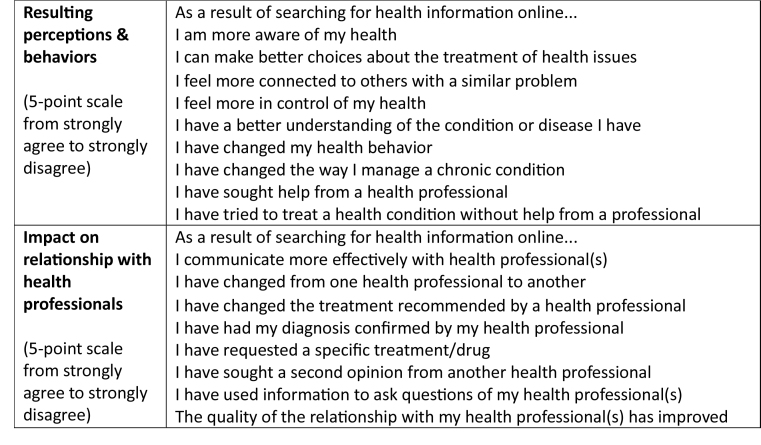


**Resulting perceptions & behaviors ** (5-point scale from strongly agree to strongly disagree)	As a result of searching for health information online...
	I am more aware of my health
	I can make better choices about the treatment of health issues
	I feel more connected to others with a similar problem
	I feel more in control of my health
	I have a better understanding of the condition or disease I have
	I have changed my health behavior
	I have changed the way I manage a chronic condition
	I have sought help from a health professional
	I have tried to treat a health condition without help from a professional

**Impact on relationship with health professionals **(5-point scale from strongly agree to strongly disagree)	As a result of searching for health information online...
	I communicate more effectively with health professional(s)
	I have changed from one health professional to another
	I have changed the treatment recommended by a health professional
	I have had my diagnosis confirmed by my health professional
	I have requested a specific treatment/drug
	I have sought a second opinion from another health professional
	I have used information to ask questions of my health professional(s)
	The quality of the relationship with my health professional(s) has improved

**Table:**
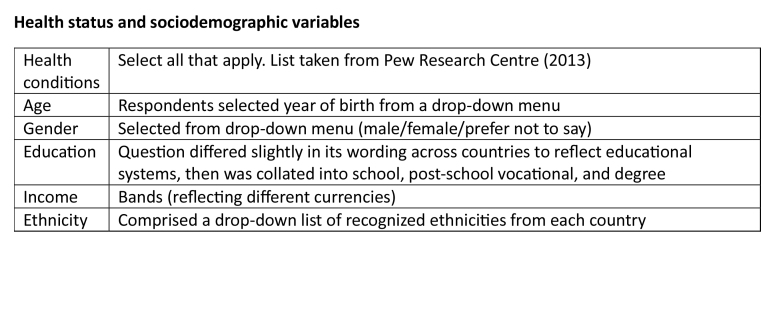


**Health status and sociodemographic variables**	
Health conditions	Select all that apply. List taken from Pew Research Centre (2013)
Age	Respondents selected year of birth from a drop-down menu
Gender	Selected from drop-down menu (male/female/prefer not to say)
Education	Question differed slightly in its wording across countries to reflect educational systems, then was collated into school, post-school vocational, and degree
Income	Bands (reflecting different currencies)
Ethnicity	Comprised a drop-down list of recognized ethnicities from each country

### Sample and Procedures

The Commonwealth Fund's country ranking on key health performance indicators (Schneider et al., 2017) guided our nation choices. We selected the top (United Kingdom) and bottom (United States) ranked. From the middle cluster we chose New Zealand because (1) it is the only non-European country; (2) aging rates are higher than other developed countries (Kowal et al., 2014); and (3) predictions suggest financial instability with current care models (Schluter et al., 2013). After obtaining full ethical approval from each of our University Ethics Committees, we commissioned commercial research organizations in each country to administer our questionnaire electronically to national random samples of baby boomers who had used the internet to search for health information in the previous 6 months. **Table B** details these procedures.

**Table B:**
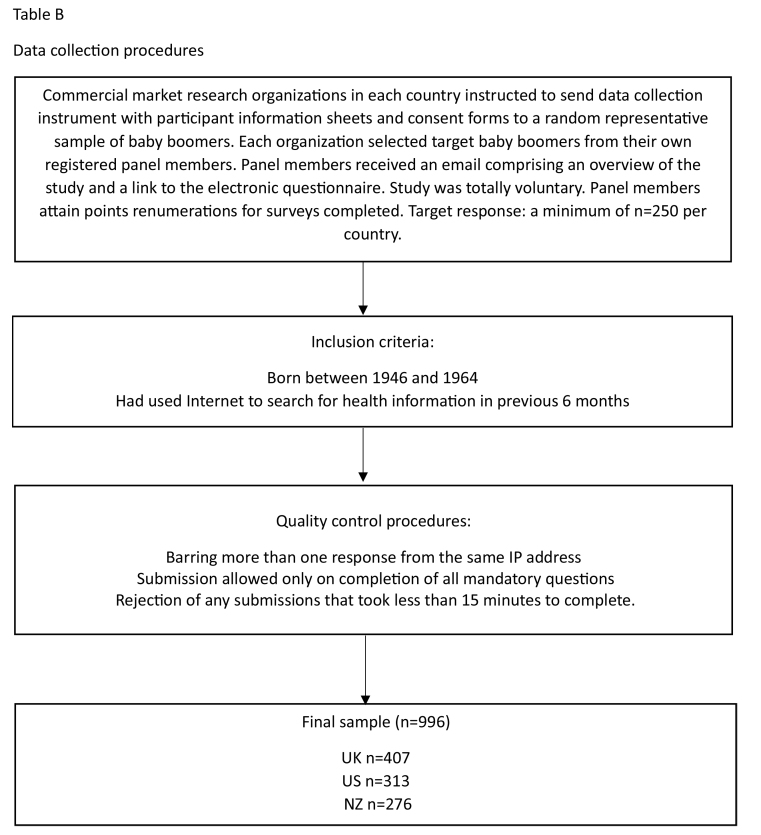
Data collection procedures

### Data Analyses

**Table 1** shows the confirmatory factor analyses (CFA) we ran to check for measurement invariance, a crucial step for multicounty data (Helsper & Gerber, 2012).

**Table 1:**
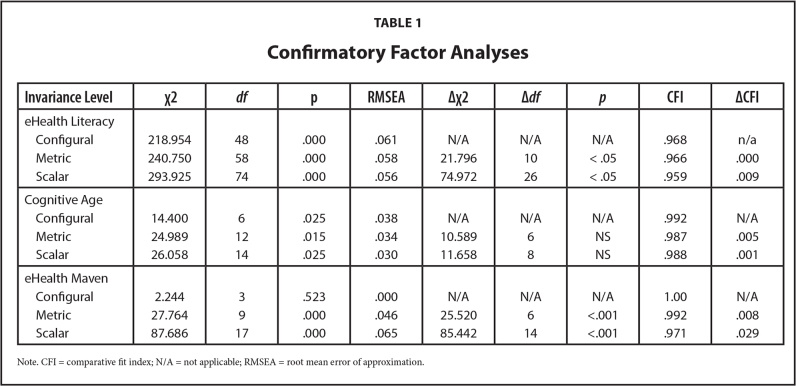
Confirmatory Factor Analyses

**Invariance Level**	**χ^2^**	***df***	**p**	**RMSEA**	**Δχ^2^**	**Δ*df***	***p***	**CFI**	**ΔCFI**

eHealth Literacy									
Configural	218.954	48	.000	.061	N/A	N/A	N/A	.968	n/a
Metric	240.750	58	.000	.058	21.796	10	< .05	.966	.000
Scalar	293.925	74	.000	.056	74.972	26	< .05	.959	.009

Cognitive Age									
Configural	14.400	6	.025	.038	N/A	N/A	N/A	.992	N/A
Metric	24.989	12	.015	.034	10.589	6	NS	.987	.005
Scalar	26.058	14	.025	.030	11.658	8	NS	.988	.001

eHealth Maven									
Configural	2.244	3	.523	.000	N/A	N/A	N/A	1.00	N/A
Metric	27.764	9	.000	.046	25.520	6	<.001	.992	.008
Scalar	87.686	17	.000	.065	85.442	14	<.001	.971	.029

Note. CFI = comparative fit index; N/A = not applicable; RMSEA = root mean error of approximation.

RMSEA results for eHealth literacy and cognitive age suggest reasonable fitting models (MacCallum et al., 1996). Both exceed minimum comparative fit index values of .95 (Hu & Bentler, 1998) and chi-square change falls below −.01 (Chen, 2007). Consequently, comparisons of latent means across groups are meaningful (Putnick & Bornstein, 2016). The CFA for eHealth literacy confirmed the three-factor structure pertaining to awareness, skills, and evaluation ability (Gartrell et al., 2020). The eHealth maven scale reached only metric invariance, so individual items are suitable for examining structural relationships with other constructs (Helsper & Gerber, 2012), but the full scale was dropped from subsequent analyses.

We then conducted cluster analysis using the non-hierarchical Euclidean distance measure (Hair et al., 2014) using the items (excluding health and sociodemographic variables) detailed in **Table A**. Different scale measurement issues were rectified by transforming variables into standardized z scores (Frades & Mattiesen, 2010). Non-hierarchical procedures demand predetermined cluster numbers, so we conducted several analyses and selected the optimum based on the distance between them and the ability to fully differentiate each. Using descriptive techniques (one way ANOVA, Chi-square, and post-hoc tests) we profiled each segment.

## Results

The final sample (*N* = 996) comprises United Kingdom (*n* = 407), US (*n* = 313), and New Zealand (*n* = 276) boomers with a mean age of 60 years drawn from an equal number of men and women. One-third were employed, almost one-third were retired, the rest comprising unemployed (*n* = 103) and homemakers (*n* = 134). In terms of education, 33% held a university degree, 35% college/professional certification, and 32% had no post-school education.

**Figure 1.**
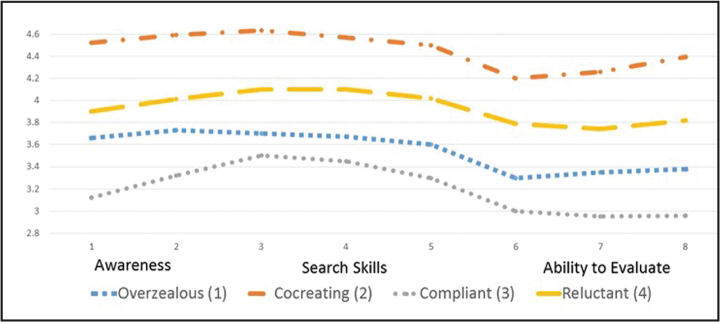
eHealth literacy by factor and segment.

Analyses identified four subgroups, profiled in **Table 2**. None are particularly healthy, which is unsurprising because boomers are less healthy than preceding generations (Davies, 2016; King et al., 2013). While the sociodemographic profiles of the segments (**Table 2**) are unremarkable, there are meaningful eHealth differences. Cognitive age failed to differentiate the segments. We gave each subgroup an epithet summarizing its characteristics: overzealous (segment 1), co-creating (segment 2), compliant (segment 3), and reluctant (segment 4). **Table C** provides detailed analyses. Noteworthy are the significant eHealth literacy differences (*F* = 236.925, *p* < .001). **Figure 1** spotlights these across the three factors (awareness of eHealth resources, accessing skills, and evaluation ability). Irrespective of segment, these boomers are less confident in their ability to evaluate eHealth information than they are in their awareness of eHealth resources and their ability to search for them.

**Table C:**
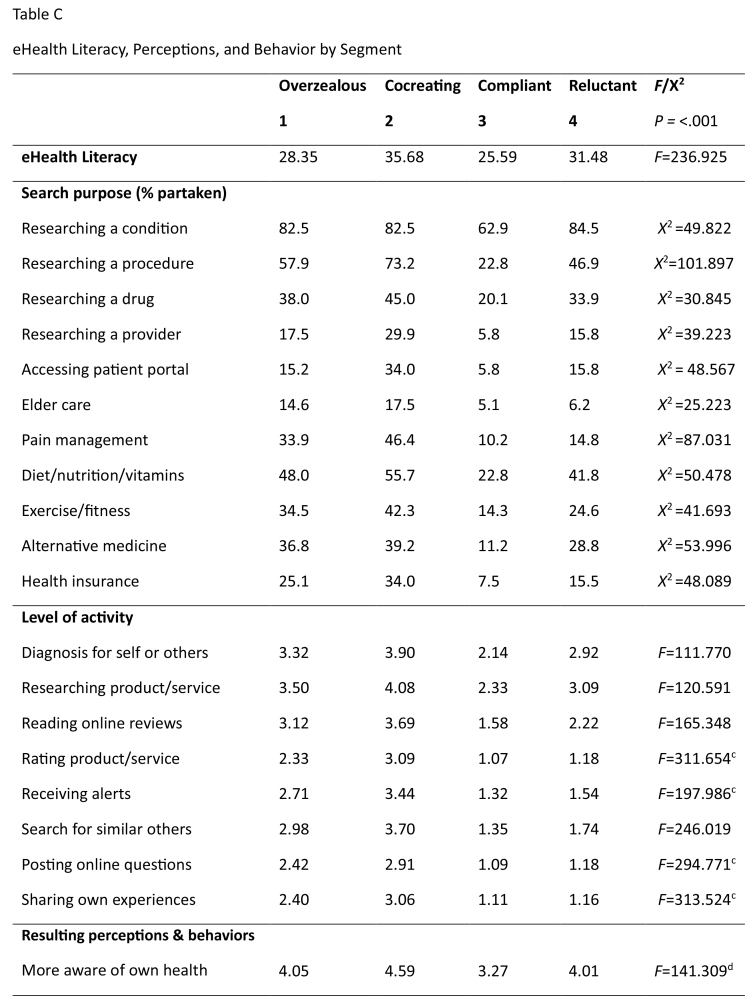
eHealth Literacy, Perceptions, and Behavior by Segment

	**Overzealous**	**Cocreating**	**Compliant**	**Reluctant**	***F*/X^2^**
	**1**	**2**	**3**	**4**	*P =* <.001

**eHealth Literacy**	28.35	35.68	25.59	31.48	*F*=236.925

**Search purpose (% partaken)**					
Researching a condition	82.5	82.5	62.9	84.5	*X*^2 ^=49.822
Researching a procedure	57.9	73.2	22.8	46.9	*X*^2^=101.897
Researching a drug	38.0	45.0	20.1	33.9	*X*^2 ^=30.845
Researching a provider	17.5	29.9	5.8	15.8	*X*^2 ^=39.223
Accessing patient portal	15.2	34.0	5.8	15.8	*X*^2 ^= 48.567
Elder care	14.6	17.5	5.1	6.2	*X*^2 ^=25.223
Pain management	33.9	46.4	10.2	14.8	*X*^2 ^=87.031
Diet/nutrition/vitamins	48.0	55.7	22.8	41.8	*X*^2 ^=50.478
Exercise/fitness	34.5	42.3	14.3	24.6	*X*^2 ^=41.693
Alternative medicine	36.8	39.2	11.2	28.8	*X*^2 ^=53.996
Health insurance	25.1	34.0	7.5	15.5	*X*^2 ^=48.089

**Level of activity**					
Diagnosis for self or others	3.32	3.90	2.14	2.92	*F*=111.770
Researching product/service	3.50	4.08	2.33	3.09	*F*=120.591
Reading online reviews	3.12	3.69	1.58	2.22	*F*=165.348
Rating product/service	2.33	3.09	1.07	1.18	*F*=311.654^c^
Receiving alerts	2.71	3.44	1.32	1.54	*F*=197.986^c^
Search for similar others	2.98	3.70	1.35	1.74	*F*=246.019
Posting online questions	2.42	2.91	1.09	1.18	*F*=294.771^c^
Sharing own experiences	2.40	3.06	1.11	1.16	*F*=313.524^c^

**Resulting perceptions & behaviors**					
More aware of own health	4.05	4.59	3.27	4.01	*F*=141.309^d^

**Table:**
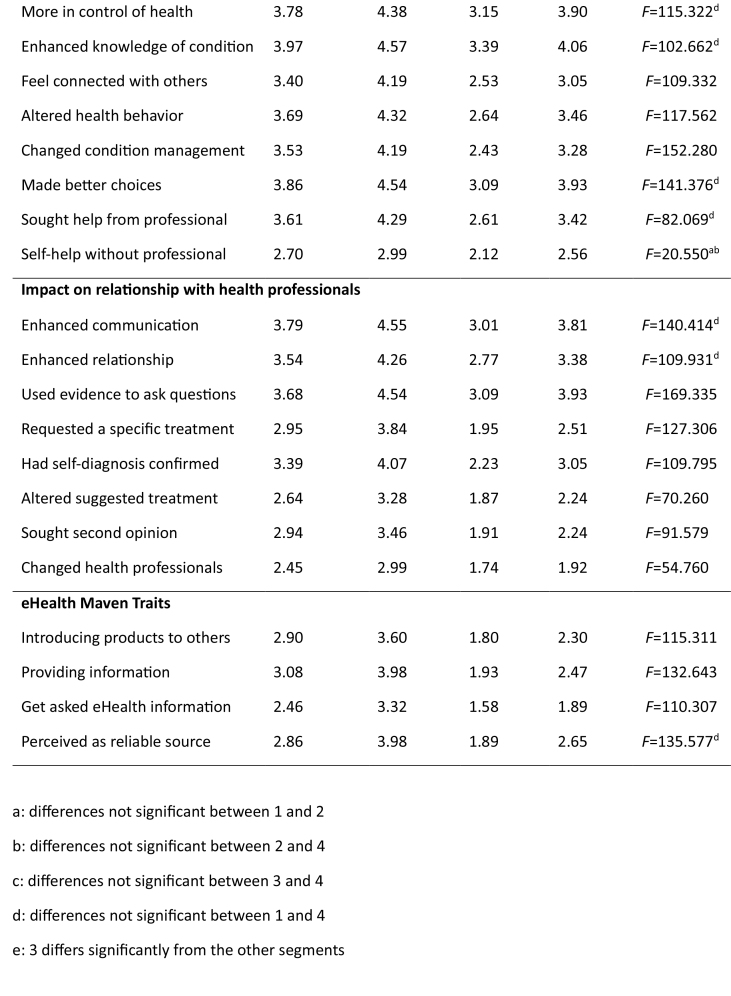


More in control of health	3.78	4.38	3.15	3.90	*F*=115.322^d^
Enhanced knowledge of condition	3.97	4.57	3.39	4.06	*F*=102.662^d^
Feel connected with others	3.40	4.19	2.53	3.05	*F*=109.332
Altered health behavior	3.69	4.32	2.64	3.46	*F*=117.562
Changed condition management	3.53	4.19	2.43	3.28	*F*=152.280
Made better choices	3.86	4.54	3.09	3.93	*F*=141.376^d^
Sought help from professional	3.61	4.29	2.61	3.42	*F*=82.069^d^
Self-help without professional	2.70	2.99	2.12	2.56	*F*=20.550^ab^

**Impact on relationship with health professionals**					
Enhanced communication	3.79	4.55	3.01	3.81	*F*=140.414^d^
Enhanced relationship	3.54	4.26	2.77	3.38	*F*=109.931^d^
Used evidence to ask questions	3.68	4.54	3.09	3.93	*F*=169.335
Requested a specific treatment	2.95	3.84	1.95	2.51	*F*=127.306
Had self-diagnosis confirmed	3.39	4.07	2.23	3.05	*F*=109.795
Altered suggested treatment	2.64	3.28	1.87	2.24	*F*=70.260
Sought second opinion	2.94	3.46	1.91	2.24	*F*=91.579
Changed health professionals	2.45	2.99	1.74	1.92	*F*=54.760

**eHealth Maven Traits**					
Introducing products to others	2.90	3.60	1.80	2.30	*F*=115.311
Providing information	3.08	3.98	1.93	2.47	*F*=132.643
Get asked eHealth information	2.46	3.32	1.58	1.89	*F*=110.307
Perceived as reliable source	2.86	3.98	1.89	2.65	*F*=135.577^d^

a: differences not significant between 1 and 2

b: differences not significant between 2 and 4

c: differences not significant between 3 and 4

d: differences not significant between 1 and 4

e: 3 differs significantly from the other segments

**Table 2:**
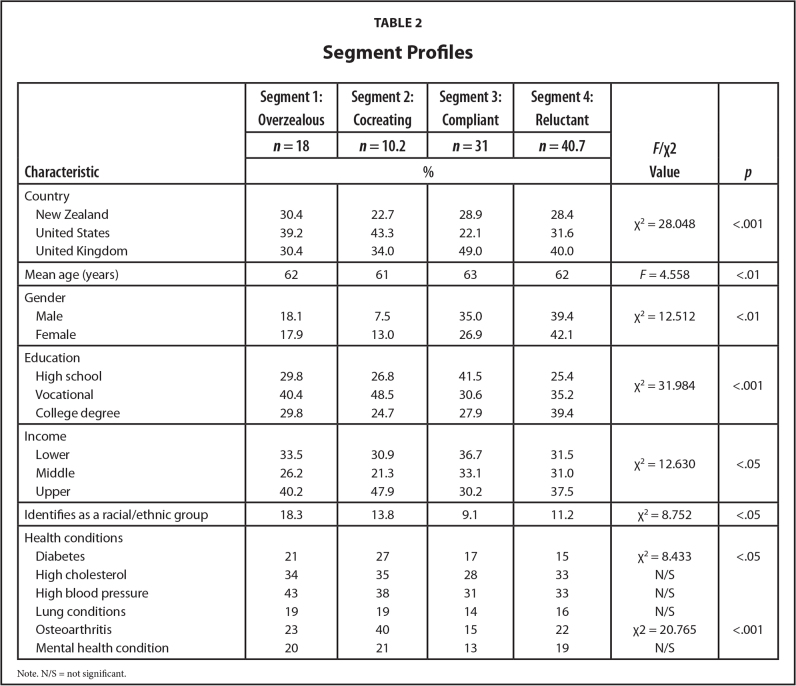
Segment Profiles

**Characteristic**	**Segment 1: Overzealous**	**Segment 2: Cocreating**	**Segment 3: Compliant**	**Segment 4: Reluctant**	***F*/χ^2^ Value**	***p***

	***n* = 18**	***n* = 10.2**	***n* = 31**	***n* = 40.7**		

	**%**					

Country						
New Zealand	30.4	22.7	28.9	28.4	χ^2^ = 28.048	<.001
United States	39.2	43.3	22.1	31.6		
United Kingdom	30.4	34.0	49.0	40.0		

Mean age (years)	62	61	63	62	*F* = 4.558	<.01

Gender						
Male	18.1	7.5	35.0	39.4	χ^2^ = 12.512	<.01
Female	17.9	13.0	26.9	42.1		

Education						
High school	29.8	26.8	41.5	25.4	χ^2^ = 31.984	<.001
Vocational	40.4	48.5	30.6	35.2		
College degree	29.8	24.7	27.9	39.4		

Income						
Lower	33.5	30.9	36.7	31.5	χ^2^ = 12.630	<.05
Middle	26.2	21.3	33.1	31.0		
Upper	40.2	47.9	30.2	37.5		

Identifies as a racial/ethnic group	18.3	13.8	9.1	11.2	χ^2^ = 8.752	<.05

Health conditions						
Diabetes	21	27	17	15	χ^2^ = 8.433	<.05
High cholesterol	34	35	28	33	N/S	
High blood pressure	43	38	31	33	N/S	
Lung conditions	19	19	14	16	Nd/S	
Osteoarthritis	23	40	15	22	χ^2^ = 20.765	<.001
Mental health condition	20	21	13	19	N/S	

Note. N/S = not significant.

### Segment 1: Overzealous

**Figure 2.**
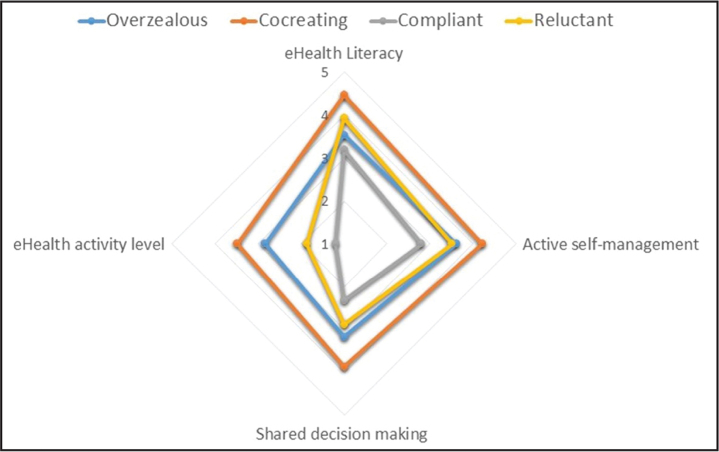
eHealth literacy and own health management by segment.

Despite possessing below average levels of eHealth literacy (**Figure 1**), this group searches frequently for eHealth information, using it for self-diagnosis and to inform decision-making with professionals (**Figure 2**). Their overzealous traits emerge from their likelihood to change their self-management of chronic conditions, often against the recommendations of health professionals. Their eHealth maven traits illustrate an eagerness to provide health information to others.

### Segment 2: Cocreating

The most eHealth literate, this subgroup comprises frequent users of different websites and online support and is significantly more likely to use social networking sites and online forums than any other. They search for a variety of information including diagnoses, drugs, and procedures, and are significantly more likely to research health care providers or use online portals to access medical results. eHealth information is used to enhance knowledge of conditions and feel connected with others, resulting in higher self-awareness and feelings of control over their own health, and changes to self-management and health behaviors. The ‘co-creating’ epithet emerges from their enhanced communications with health practitioners. They also like to share eHealth information with others.

### Segment 3: Compliant

This group is opposite to the cocreating. They have the lowest eHealth literacy levels and while all used the internet for eHealth purposes in the previous 6 months, this segment comprises extremely low users who engage infrequently with electronic resources, usually to gain information about a particular condition. Consequently, the impact of eHealth information on their perceptions, behavior, and interaction with others is minimal, making them compliant and passive recipients of health care.

### Segment 4: Reluctant

Despite relatively high levels of eHealth literacy, this group is as unlikely as the passive segment to use eHealth. Their reluctance stems particularly from engaging with similar others, sharing information, and participating in online reviews.

This well-educated segment has considerable skills to seek, find, and appraise eHealth information, using it to better understand conditions and interact with health care providers. However, they display passive tendencies in their reluctance to request or alter treatments or seek second opinions. **Figure 2** spotlights the need to manage their own health more actively.

## Discussion

In answer to our research questions, results demonstrate that there are four distinct identifiable eHealth literacy segments (research question 1), all of which use the internet for eHealth purposes. Across the segments, there are notable differences in the information sources they access and the underlying reasons for access (research question 2). Health perceptions and behaviors, including relationships with health practitioners, differ based on the eHealth information they gather (research question 3). Finally, we identify a limited range of further psychographic variables that differentiate the segments (research question 4) in that while they do not differ in terms of cognitive age, their eHealth Maven traits of sharing and providing others with health information are meaningful.

These groups are not markedly different from a sociodemographic perspective, which contrasts with much previous work (Kontos et al., 2014; Wynn et al., 2020). Interestingly, Arcury et al.'s (2020) research is the only known study that, like ours, found sociodemographic differences did not account for eHealth literacy levels among those who use the Internet for health purposes. Hence, when samples are limited to older internet users, as opposed to users and non-users, different and more nuanced patterns emerge. This does not suggest that studies examining sociodemographic or socioeconomic determinants of eHealth, or the digital divide, are unhelpful. Rather, they are crucial to evidence the ways digitalization of information reinforces existing social inequalities (Azzopardi-Muscat & Sørensen, 2019). However, because so few studies have examined differences within older populations who do use eHealth technologies, key and previously unidentified differences have hitherto remained hidden. Failing to take account of key differences within older populations means that the established body of work, which demonstrates unequivocally that age and eHealth are negatively correlated, has resulted, unintentionally, in older adults tending to be treated as a homogenous mass. At best, there is a recognition that different generational cohorts (for example baby boomers contrasting with their predecessors the silent generation) should be considered (Alvarez-Galvez et al., 2020). In contrast, our results reveal that there are four very different groups within this single generational cohort, each of which has different needs and would benefit from different interventions and eHealth strategies.

The cocreating segment (segment 2) is relatively competent in terms of eHealth literacy and uses this to advance their own health behaviors as well as sharing information with others. Noteworthy, however, is that this segment comprises only 10% of the sample. Our sample excluded people who had not accessed eHealth in the previous 6 months, suggesting that the actual number of baby boomers who fall outside this segment, and who need intervention to improve their eHealth literacy, is substantial. Indeed, even within this segment of relatively competent, relatively frequent users of eHealth technologies, only one-third had used a patient portal in the previous 6 months. Patient portals are integral to personalized care and health care cost reduction, becoming mandated by the Centers for Medicare and Medicaid (Arcury et al., 2017), and are increasingly used by the National Health Services in the United Kingdom and New Zealand (Health Navigator, 2023; NHS Digital, 2023). Clearly, non-use of patient portals and other eHealth applications risks the implications of digital divide becoming greater.

Given the well documented poor health outcomes that result from low health literacy (Berard et al., 2020), coupled with the acceleration of eHealth across many nations (WHO, 2020), policy interventions and education are needed for all baby boomers. Of the three factors that make up our chosen eHealth literacy measure (Norman & Skinner, 2006), the ability to evaluate online health information is lower across all segments, which is of particular concern when one considers search engine optimization (Schultheiß et al., 2022), the algorithms used to provide users with online information (Gruber & Hargittai, 2023), and the omnipresence of online misinformation that is potentially harmful (Wagner et al., 2022). What is of particular significance from these results, however, is that different segments require different interventions. Assessment of eHealth literacy levels should immediately follow diagnosis of a chronic disease. Certainly, there are available valid and reliable instruments that are easy and relatively quick to administer (see Karnoe & Kayser, 2015, for a review), and we found the eHealth literacy scale (Norman & Skinner, 2006) particularly easy to use and understand. Hence, clinician burden would not be onerous. Signposting patients toward the right support available to them needs to follow.

## Study Limitations

Although carefully selected, this research is limited to only three national samples, suggesting opportunities for future research to incorporate greater numbers of nations and cultures. We also selected baby boomers who already use eHealth information. Continued research needs to incorporate nonusers to identify barriers to adoption of eHealth. This is particularly important if, as is widely suggested, eHealth provides an opportunity to promote and facilitate health and wellbeing (WHO, 2020). Additionally, the study is limited to a self-complete online questionnaire with their well-documented limitations (see Evans & Mathur, 2005 for an in-depth review). Our carefully designed data collection procedures (see **Table B**) hopefully mitigated drawbacks such as perceptions of junk mail and privacy issues, and our use of representative samples overcame the tendency for online samples to be skewed. Moreover, the chosen eHealth literacy measure does rely on subjective self-assessment (Norman & Skinner, 2006). Nevertheless, objective validation of reported eHealth literacy levels and indeed the ways in which the different segments behave and interact with health care providers would add validity to the study.

As with any segmentation model, our segments reflect a snapshot in time; they capture the current situation (Docters et al., 1997). But segments comprise people, so as people change so do segments. Our segments are meant as a useful starting point for evidence-based plans for the different interventions needed to better meet the needs of the future older individuals. Of course, not all providers are motivated by the new personalized care paradigm that argues that patients are better served by understanding what is important for the individual as a person, not just a patient with a condition, and by facilitating discussions and shared decision making and planning (Royal College of Physicians, 2018). However, it will be useful to those who do wish to engage.

## Conclusion

Aging populations, rising health care costs, increasing morbidity, and recovery from the pandemic are pressurizing health care systems. eHealth is frequently heralded as having the potential to reduce costs, improve care quality, enhance patient empowerment, and encourage participation in health self-management (WHO, 2020). However, to use electronic sources effectively, patients need to be eHealth literate. The consistent finding that eHealth literacy and age are negatively related (Hsu, 2019) has resulted in older adults being classified as a homogenous mass. This research spotlights a more complex picture and finds that within an older cohort of baby boomers there are four very different subgroups, each of which require tailored strategies to encourage effective use of eHealth resources for future planning, given the unprecedented demands this cohort is predicted to place on many health care systems across the world.
